# Factors Associated With Family Member’s Spanking of 3.5-year-old Children in Japan

**DOI:** 10.2188/jea.JE20190160

**Published:** 2020-10-05

**Authors:** Sachiko Baba, Ehab S. Eshak, Kokoro Shirai, Takeo Fujiwara, Yui Yamaoka, Hiroyasu Iso

**Affiliations:** 1 Bioethics and Public Policy, Department of Social Medicine, Osaka University Graduate School of Medicine, Osaka, Japan; 2 Public Health, Department of Social Medicine, Osaka University Graduate School of Medicine, Osaka, Japan; 3 Department of Global Health Promotion, Tokyo Medical and Dental University, Tokyo, Japan; 4 University of Oklahoma Health Sciences Center, Oklahoma City, OK, USA

**Keywords:** corporal punishment, spanking, parenting, socioeconomic factor, family structure

## Abstract

**Background:**

Spanking can cause adverse psychological development and biological functional changes in children. However, spanking is widely used by parents in Japan. This study explored the risk factors for family member’s spanking of 3.5-year-old children using nationwide population data in Japan.

**Methods:**

Surveys were administered to family members in Japan who had a child in 2001 (first cohort) or in 2010 (second cohort), and the data when their child was 0.5, 1.5, and 3.5 years old were used. We used multivariate binary and ordinal logistic regression analyses to examine the associations between risk factors and spanking children at 3.5 years of age, which was subcategorized into frequencies of never, sometimes, and always spanking, presented with odds ratios (ORs) and 95% confidence intervals (CIs).

**Results:**

Among 70,450 families, 62.8% and 7.9% sometimes and always spanked their children, respectively. Children in the second cohort were spanked less frequently compared with those in the first cohort, and fathers who responded to the questionnaire spanked children less frequently than mothers who responded. Identified associated factors for spanking were male child, presence of siblings of the child, not living in a two-parent household, not living in a three-generation household, younger parents, parents with lower education, no outside work or unstable work, and lower family income.

**Conclusions:**

We found a high prevalence of spanking and its associated factors. Approaching those with lower socioeconomic factors and promoting fathers’ involvement in parenting may be important public health strategies for reducing and preventing spanking.

## INTRODUCTION

Spanking is the most common form of corporal punishment.^1^ More than 50 countries worldwide have banned corporal punishment because, regardless of its severity, it is known to be associated with physical abuse,^2^ adverse psychological development, and biological changes in neural functioning.^2^^–^^5^ Previous studies have shown behavioral problems among spanked children, including external aggression or antisocial behavior, and impaired mental health problems, such as suicide.^4^^,^^6^^–^^10^

The United Nations enacted the Convention on the Rights of the Child to protect children from corporal punishment and other cruel or degrading forms of punishment in 1989.^11^ However, corporal punishment remains a relatively common disciplinary measure in Japan and other countries worldwide.^12^ According to a recent survey among Japanese, approximately 70% of parents experienced being spanked as a child and 60% admitted to spanking their own children.^13^ This reflects the fact that corporal punishment by parents had not been banned, whereas corporal punishment by school teachers has been banned in Japan since 1947 under the School Education Act (Act No. 26).^14^ Legislative approaches are reported to be a promising strategy in order to reduce physical abuse of children in other countries.^15^ In June 2019, the Japanese Diet enacted amendments to the Child Welfare Act (Act No. 164 of 1947)^16^ and the Act on the Prevention, etc of Child Abuse (Act No. 82 of 2000),^17^ including a ban on corporal punishment of children by parents and other guardians, which will go into effect in April 2020.^18^ Therefore, it is of the utmost importance to provide scientific evidence on the risk factors of spanking and corporal punishment among the Japanese population.

Identified risk factors for spanking and corporal punishment consist of parent, child, family, and community/cultural factors.^1^^,^^2^^,^^9^ Reported parental factors from other countries are as follows: very young mothers, lower socioeconomic status (SES), poor maternal physical and mental health, personal experience of physical discipline or abuse, and elevated parenting stress.^1^^,^^10^^,^^19^^–^^22^

However, as far as we know, there has been no large epidemiological study in Japan that broadly examined the associations between risk factors and parents’ spanking. Moreover, there have been no studies to observe the transition in use of parents’ spanking in different generations in countries where corporal punishment has not been banned. Therefore, the purpose of this study was to explore the parental risk factors of spanking of 3.5-year-old children using nationwide population data in Japan.

## MATERIAL AND METHODS

### Study participants

Study data were obtained from a Japanese panel survey entitled, “The Longitudinal Survey on Babies Born in the 21^st^ Century”. Based on vital statistics birth record lists, the study sample of the first-generation cohort included all participants whose children were born in Japan between January 10 and 17, 2001 or between July 10 and 17, 2001 (*n* = 53,575). The second-generation cohort included all participants whose children were born in Japan between May 10 and 24, 2010 (*n* = 43,767). These selected participants were recruited via mail questionnaires sent to the children’s residence when the children were 0.5 years old, which corresponded to the first-wave panel survey. Respondents were considered to have agreed to participate in the study if the questionnaire was returned to the Ministry of Health, Labour and Welfare. There were 47,015 responses (response rate of 87.8%) for the first cohort and 38,554 responses (response rate of 88.1%) for the second cohort, for a total sample of 85,569 in the first-wave panel survey. The surveys were conducted annually and included approximately 20 questions. The variables used in this study were retrieved from data gathered from the first- (0.5 years old), second- (1.5 years old), and fourth- (3.5 years old) wave panel surveys in each cohort. Participants who had not provided information on spanking at the fourth wave (ie, when their children were 3.5 years old) survey were excluded (*n* = 15,119), which led to a final sample size of 70,450 for this study.

### Outcome

Family members were asked in the fourth-wave survey (in 2003–2004 for the first cohort, and in 2013 for the second cohort), when their children were 3.5 years old, “How do you generally react to your child’s misbehavior?”. The response choices were as follows: 1) explaining why the behavior was wrong, 2) saying “No” without any explanation, 3) spanking, 4) allowing them to recognize their misbehavior by ignoring them, and 5) making them go outside or putting them in a closet. Family members were required to answer each item by selecting the frequency from the three choices of always, sometimes, and never. Spanking was used as the outcome for this study.

### Exposure

Respondents in the fourth-wave survey were asked to report their relationship to the child (ie, mother, father, maternal grandmother, maternal grandfather, paternal grandmother, paternal grandfather, or other). Multiple responses were allowed. We re-categorized the respondent as mother, father, both parents, and other family members in this study. Other family members were those who did not include either parent as a respondent. For example, if mother and maternal grandmother were chosen, it was re-categorized as “mother”, whereas if only the maternal grandmother was chosen, it was re-categorized as “other family members”. Caregivers were defined using the question “Who usually takes care of the child?”, with the following choices of answers: mother, father, maternal grandmother, maternal grandfather, paternal grandmother, paternal grandfather, nursery teachers, babysitters, kindergarten teachers, and others. Multiple answers were allowed. We re-categorized the responses as mother, father, both parents, and others in this study. Others were those who did not include either parent as a caregiver. For example, if mother and nursery teachers were chosen, it was re-categorized as “mother”, whereas if only nursery teachers were chosen, it was re-categorized as “others”. Information about potential risk factors for spanking was also collected, including the gender of the child, family structure (presence of siblings of the child, living in a two-parent household, and living in a three-generation household), and family socioeconomic factors (parents’ age, level of education, working hours, work type, and family income). Information on the parents’ level of education was obtained from the second-wave survey, and family income, presence of siblings of the child, living in a two-parent household, living in a three-generation household, and parents’ working hours and work types were obtained from the fourth-wave survey.

Gender of the child was categorized as either boy or girl. The presence of siblings of the child was dichotomously re-categorized. For the variable “living in a two-parent household”, a parent was defined as such irrespective of whether it was the biological parent or a step-parent. Living in a three-generation household was defined as child living with either the mother and/or father and one or more grandparents.

Parents’ age was calculated according to the parents’ birth date information obtained from the first-wave survey, and categorized into six age groups. Parent’s level of education was obtained by asking their highest level of education and was categorized as junior high school, high school, vocational school, junior college, university and higher, and others. Parents’ working hours per week was categorized as 0 hours, 1–19 hours, 20–39 hours, and 40 hours and over. Parents’ work type was categorized as no outside work (ie, housewife), seeking employment, student, employed full-time, employed part-time, self-employed, domestic side job, and others. For the variables where parents’ characteristics were provided and the respondents were “both parents”, the mother’s variables were used because mothers comprised more than 90% of the participants in this study.

Family income was calculated by summing the mother’s and father’s incomes during the last year as obtained from the fourth-wave survey. If the income for either parent was missing, the other parent’s income was considered the family income. Family income was re-categorized into quartiles. Responses of always using forms of discipline other than spanking were used as a covariate.

We obtained permission to use the panel survey data from the Ministry of Health, Labour and Welfare under the Statistics Act in Japan (No. 1020-3). This study was approved by the Ethics Review Board of Osaka University Hospital (No. 16154).

### Statistical analyses

The proportion of the frequencies of always, sometimes, and never spanking were calculated for each category of survey respondents, caregivers, gender of the child, presence of siblings of the child, living with the child’s father, living in a three-generation household, parents’ age, parents’ level of education, parents’ working hours, parents’ work type, family income, and cohort generation. The statistical significance of the differences in these proportions was analyzed using the χ^2^ test.

Binary logistic regression analyses were used to examine the associations between potential risk factors and spanking. We estimated the risk of sometimes and always spanking, respectively, by obtaining odds ratios (ORs) and their corresponding 95% confidence intervals (CIs) in comparison to never spanking. We also used ordinal logistic regression analyses to estimate the association between potential risk factors and cumulated risk of sometimes or always spanking in an ordered manner. We further applied ordinal logistic regression analyses, stratified by cohort generation and the gender of the child, respectively. All analyses were performed using Statistical Analysis Software version 9.4 (SAS Institute, Inc., Cary, NC, USA).

## RESULTS

Of the total 70,450 respondents, 88.5% (*n* = 62,349) were mothers, 5.9% (*n* = 4,140) were fathers, 4.7% (*n* = 3,290) were both parents, and 1.0% (*n* = 671) were other family members. A total of 45.3% (*n* = 31,894) of caregivers were mothers, 0.2% (*n* = 174) were fathers, 46.8% (*n* = 32,972) were both parents, and 7.7% (*n* = 5,410) were others (Table 1). Most respondents in the category of other family members were maternal or paternal grandmothers, and most caregivers in the category others were nursery or kindergarten teachers (data not shown). Distributions of spanking and potential risk factors are shown in Table 2. Among all participants, 62.8% sometimes spanked, and 7.9% always spanked to discipline the child at age 3.5 years old. The proportions of always spanking were higher in the following categories: the first cohort; child gender of boy; presence of siblings of the child; not living in a two-parent household or living in a three-generation household; younger parents; parents with a lower level of education or shorter working hours; mother having no outside work, seeking employment, or having a domestic side job; father seeking employment; and lower family income.

**Table 1.** Distribution of respondents of the survey and caregivers

Respondents	Caregivers				

	Mother	Father	Both	Others	Total
Mother	29,350	52	28,238	4,709	62,349
Father	1,386	89	2,258	407	4,140
Both	896	2	2,247	145	3,290
Other family members	262	31	229	149	671
Total	31,894	174	32,972	5,410	70,450

**Table 2.** Characteristics of participants

	Total number	Spanking						

		Never		Sometimes		Always		*P*-value
			
	*n*	*n*	Proportion	*n*	Proportion	*n*	Proportion	
Total	70,450	20,581	29.2	44,271	62.8	5,598	7.9	<0.01
								
Respondent of the survey								
Mother	62,349	17,582	28.2	39,672	63.6	5,095	8.2	<0.01
Father	4,140	1,587	38.3	2,280	55.1	273	6.6	
Both	3,290	1,198	36.4	1,910	58.1	182	5.5	
Other family members	671	214	31.9	409	61.0	48	7.2	
Caregiver of the child								
Mother	31,894	8,933	28.0	20,218	63.4	2,743	8.6	<0.01
Father	174	63	36.2	102	58.6	9	5.2	
Both	32,972	9,952	30.2	20,655	62.6	2,365	7.2	
Others	5,410	1,633	30.2	3,296	60.9	481	8.9	
Cohort generation								
First	41,193	9,391	22.8	27,727	67.3	4,075	9.9	<0.01
Second	29,257	11,190	38.2	16,544	56.5	1,523	5.2	
Gender of the child								
Boy	36,465	9,109	25.0	23,907	65.6	3,449	9.5	<0.01
Girl	33,985	11,472	33.8	20,364	59.9	2,149	6.3	
Presence of siblings of the child								
Yes	53,534	14,478	27.0	34,564	64.6	4,492	8.4	<0.01
Living in a two-parent household								
No	3,300	908	27.5	2,056	62.3	336	10.2	<0.01
Living in a three-generation household								
Yes	12,756	3,915	30.7	7,756	60.8	1,085	8.5	<0.01
								
Mother’s age, years								
<25	1,377	320	23.2	858	62.3	199	14.5	<0.01
25–29	10,545	2,481	23.5	6,824	64.7	1,240	11.8	
30–34	27,867	7,759	27.8	17,827	64.0	2,281	8.2	
35–39	22,649	7,088	31.3	14,094	62.2	1,467	6.5	
40–44	7,336	2,675	36.5	4,279	58.3	382	5.2	
≥45	676	258	38.2	389	57.5	29	4.3	
Father’s age, years								
<25	810	205	25.3	502	62.0	103	12.7	<0.01
25–29	7,219	1,674	23.2	4,689	65.0	856	11.9	
30–34	22,023	5,920	26.9	14,178	64.4	1,925	8.7	
35–39	23,812	7,272	30.5	14,851	62.4	1,689	7.1	
40–44	12,163	4,034	33.2	7,386	60.7	743	6.1	
≥45	4,423	1,476	33.4	2,665	60.3	282	6.4	
Mother’s education								
Junior high school	2,268	540	23.8	1,438	63.4	290	12.8	<0.01
High school	22,952	5,464	23.8	15,133	65.9	2,355	10.3	
Vocational	13,388	3,591	26.8	8,683	64.9	1,114	8.3	
Junior college	16,082	4,874	30.3	10,142	63.1	1,066	6.6	
University and higher	13,539	5,435	40.1	7,510	55.5	594	4.4	
Others	120	50	41.7	64	53.3	6	5.0	
Missing	2,101	627	29.8	1,301	61.9	173	8.2	
Father’s education								
Junior high	3,896	882	22.6	2,553	65.5	461	11.8	<0.01
High school	23,901	6,029	25.2	15,591	65.2	2,281	9.5	
Vocational	9,936	2,610	26.3	6,472	65.1	854	8.6	
Junior college	2,252	664	29.5	1,415	62.8	173	7.7	
University and higher	27,418	9,495	34.6	16,357	59.7	1,566	5.7	
Others	139	44	31.7	87	62.6	8	5.8	
Missing	2,908	857	29.5	1,796	61.8	255	8.8	
Mother’s working hours								
0 hours	39,276	10,860	27.7	25,147	64.0	3,269	8.3	<0.01
1–19 hours	6,663	1,874	28.1	4,225	63.4	564	8.5	
20–39 hours	13,557	4,114	30.3	8,434	62.2	1,009	7.4	
≥40 hours	9,592	3,353	35.0	5,613	59.4	626	6.5	
Missing	1,362	380	27.9	852	62.6	130	9.5	
Father’s working hours								
0 hours	704	188	26.7	447	63.5	69	9.8	<0.01
1–19 hours	1,081	289	26.7	649	60.0	143	13.2	
20–39 hours	4,468	1,267	28.4	2,803	62.7	398	8.9	
≥40 hours	58,931	17,364	29.5	37,083	59.4	4,484	7.6	
Missing	5,266	1,473	28.0	3,289	62.5	504	9.6	
Mother’s work type								
No outside work	33,616	9,043	26.9	21,754	64.7	2,819	8.4	<0.01
Seeking employment	3,264	983	30.1	1,986	60.8	295	9.0	
Students	118	31	26.3	77	61.4	10	8.5	
Employed full-time	13,328	4,792	36.0	7,726	58.0	810	6.1	
Employed part-time	13,580	3,827	28.2	8,641	63.6	1,112	8.2	
Self-employed	3,529	1,057	30.0	2,204	62.4	268	7.6	
Domestic side job	1,141	269	23.6	749	65.6	123	10.8	
Others	739	229	31.0	438	59.3	72	9.7	
Missing	1,135	350	30.8	696	63.4	89	7.8	
Father’s work type								
No outside work	79	21	26.6	53	67.1	5	6.3	<0.01
Seeking job	520	131	25.2	330	63.5	59	11.3	
Students	51	17	33.3	33	61.4	1	2.0	
Full-time	57,036	16,838	29.5	35,808	62.8	4,390	7.7	
Part-time	808	222	27.5	510	63.1	76	9.4	
Self-employed	8,179	2,250	27.5	5,218	62.4	711	8.7	
Domestic side job	1	1	100.0	0	0.0	0	0.0	
Others	524	145	27.7	326	62.2	53	10.1	
Missing	3,252	956	29.4	1,993	63.4	303	9.3	
Family income (1,000,000 JPY)								
Lowest quantile (0–<3.8)	15,978	4,015	25.1	10,357	64.8	1,606	10.1	<0.01
Second lowest quantile (3.8–4.99)	13,797	3,592	26.0	9,005	65.3	1,200	8.7	
Second highest quantile (5.0–6.99)	19,056	5,547	29.1	12,083	63.4	1,426	7.5	
Highest quantile (≥7.0)	16,887	6,070	35.9	9,884	58.5	933	5.5	
Missing	4,732	1,357	28.7	2,942	62.2	433	9.2	
“Always” use of other forms of disciplines								
Explaining why this behavior was wrong	58,561	17,515	29.9	36,719	62.7	4,327	7.4	<0.01
Saying “No” without any explanation	15,186	2,893	19.1	9,707	63.9	2,586	17.0	<0.01
Allowing them to recognize their misbehavior by ignoring them	981	182	18.6	489	49.8	310	31.6	<0.01
Letting them go out or put in a closet	328	29	8.8	114	34.8	185	56.4	<0.01

Table 3 shows the associations between each risk factor and spanking in the binary and ordinal logistic regressions. In terms of respondents, compared with the mother, the father or both parents had lower risk, while others had higher risk of spanking in the ordinal logistic regression model: adjusted ORs were 0.85 (95% CI, 0.79–0.92), 0.92 (95% CI, 0.86–0.99), and 1.34 (95% CI, 1.13–1.59) respectively. Compared with the first cohort, the second cohort showed lower odds for spanking: the adjusted ORs in the binary logistic regression model were 0.67 (95% CI, 0.65–0.70) for sometimes spanking and 0.58 (95% CI, 0.54–0.62) for always spanking, and that obtained from the ordinal logistic regression analyses was 0.54 (95% CI, 0.52–0.56). The gender of the child was associated with spanking: the adjusted ORs for spanking boys in the binary logistic regression were 1.25 (95% CI, 1.21–1.29) for sometimes spanking and 1.45 (95% CI, 1.37–1.54) for always spanking, and the adjusted OR for spanking boys was 1.48 (95% CI, 1.43–1.52) in the ordinal logistic regression model. For family structure factors, the presence of a sibling of the child and not living with both parents were associated with spanking, while living within a three-generation household was inversely associated with spanking. For family socioeconomic factors, dose-response inverse associations of parents’ age, parents’ level of education, and family income with spanking were observed. Compared with parents who were employed full-time, parents who had no outside work, were employed part-time, were self-employed, or had a domestic side job were more likely to spank their children: the adjusted ORs in the ordinal logistic regression model were 1.21 (95% CI, 1.10–1.33), 1.10 (95% CI, 1.03–1.17), 1.11 (95% CI, 1.02–1.20), 1.19 (95% CI, 1.04–1.37), respectively. In the sensitivity analysis where adjusted ORs were examined when stratified by respondents, respondents did not modify the associations between caregivers and spanking (data not shown). In the same manner, the sensitivity analysis where adjusted ORs were examined when stratified by caregivers, caregivers did not modify the associations between respondents and spanking (data not shown).

**Table 3.** Adjusted^a^ odds ratios for spanking by binary and ordinal logistic regression model

	Binary logistic regression						Ordinal logistic regression		

	Sometimes spanking			Always spanking					
		
	aOR	(95% CI)	Trend *P*	aOR	(95% CI)	Trend *P*	aOR	95% CI	Trend *P*
Respondents of the survey									
Mother	Reference		0.003	Reference		0.62	Reference		0.0008
Father	0.86	(0.80–0.93)		1.04	(0.89–1.22)		0.85	(0.79–0.92)	
Both	0.96	(0.89–1.03)		0.89	(0.89–1.22)		0.92	(0.86–0.99)	
Other family members	1.25	(1.05–1.48)		1.33	(0.94–1.88)		1.34	(1.13–1.59)	
Caregiver of the child									
Mother	Reference		0.70	Reference		<0.0001	Reference		<0.0001
Father	0.95	(0.69–1.30)		0.63	(0.31–1.26)		0.75	(0.55–1.03)	
Both	1.03	(0.99–1.06)		0.90	(0.84–0.96)		0.97	(0.94–1.01)	
Others	0.96	(0.90–1.02)		0.98	(0.87–1.10)		0.95	(0.89–1.01)	
Cohort generation									
First	Reference		<0.0001	Reference		<0.0001	Reference		<0.0001
Second	0.67	(0.65–0.70)		0.58	(0.54–0.62)		0.54	(0.52–0.56)	
Gender of the child									
Boy	1.25	(1.21–1.29)	<0.0001	1.45	(1.37–1.54)	<0.0001	1.48	(1.43–1.52)	<0.0001
Girl	Reference			Reference			Reference		
Presence of siblings of the child									
No	Reference		<0.0001	Reference		<0.0001	Reference		<0.0001
Yes	1.31	(1.26–1.36)		1.21	(1.12–1.30)		1.40	(1.35–1.45)	
Living in a two-parent household									
No	1.11	(1.03–1.21)	0.19	1.16	(1.01–1.33)	0.005	1.19	(1.10–1.29)	<0.0001
Yes	Reference			Reference			Reference		
Living in a three-generation household									
No	1.2	(1.15–1.25)	<0.0001	1.09	(1.01–1.18)	0.07	1.24	(1.19–1.30)	<0.0001
Yes	Reference			Reference			Reference		
Parent’s age, years									
<25	0.9	(0.79–1.02)	<0.0001	1.34	(1.11–1.60)	<0.0001	1.15	(1.02–1.30)	<0.0001
25–29	0.97	(0.92–1.02)		1.26	(1.16–1.36)		1.14	(1.09–1.20)	
30–34	Reference			Reference			Reference		
35–39	1.00	(0.96–1.04)		0.92	(0.86–0.99)		0.97	(0.93–1.00)	
40–44	0.96	(0.84–1.09)		0.81	(0.72–0.92)		0.87	(0.82–0.92)	
≥45	0.97	0.85 1.10		0.74	(0.55–1.00)		0.88	(0.78–1.01)	
Parent’s education									
Junior high school	1.18	(1.07–1.31)	0.09	1.84	(1.56–2.17)	<0.0001	1.59	(1.44–1.76)	<0.0001
High school	1.26	(1.21–1.33)		1.66	(1.51–1.84)		1.54	(1.47–1.62)	
Vocational	1.32	(1.25–1.39)		1.48	(1.33–1.65)		1.48	(1.40–1.55)	
Junior college	1.21	(1.16–1.28)		1.24	(1.11–1.38)		1.27	(1.21–1.33)	
University and higher	Reference			Reference			Reference		
Others	1.06	(0.73–1.54)		0.66	(0.23–1.84)		0.92	(0.63–1.34)	
Missing	1.21	(1.09–1.33)		1.48	(1.22–1.79)		1.37	(1.24–1.52)	
Parent’s working hours									
0 hour	1.00	(0.90–1.10)	0.55	1.07	(0.87–1.32)	0.03	1.02	(0.92–1.12)	0.003
1–19 hours	1.02	(0.94–1.11)		1.04	(0.89–1.21)		1.04	(0.96–1.12)	
20–39 hours	1.03	(0.97–1.10)		0.98	(0.87–1.11)		1.02	(0.95–1.08)	
≥40 hours	Reference			Reference			Reference		
Missing	0.92	(0.79–1.08)		1.04	(0.78–1.38)		0.96	(0.81–1.12)	
Parent’s work type									
No outside work	1.17	(1.06–1.28)	0.44	1.08	(0.89–1.32)	0.64	1.21	(1.10–1.33)	0.54
Seeking employment	1.02	(0.90–1.15)		1.14	(0.91–1.45)		1.10	(0.98–1.24)	
Students	1.16	(0.77–1.75)		1.13	(0.53–2.42)		1.18	(0.78–1.78)	
Employed full-time	Reference			Reference			Reference		
Employed part-time	1.09	(1.02–1.16)		1.04	(0.92–1.18)		1.10	(1.03–1.17)	
Self-employed	1.07	(0.99–1.16)		1.09	(0.93–1.28)		1.11	(1.02–1.20)	
Domestic side job	1.08	(0.93–1.24)		1.24	(0.98–1.57)		1.19	(1.04–1.37)	
Others	1.05	(0.89–1.23)		1.37	(1.02–1.83)		1.23	(1.04–1.45)	
Missing	1.19	(0.99–1.42)		1.00	(0.71–1.40)		1.15	(0.97–1.38)	
Family income (1,000,000 JPY)									
Lowest quantile (0≤, <3.8)	1.15	(1.10–1.22)	<0.0001	1.22	(1.11–1.35)	<0.0001	1.24	(1.18–1.31)	<0.0001
Second lowest quantile (3.8–4.99)	1.18	(1.12–1.25)		1.18	(1.07–1.31)		1.25	(1.19–1.31)	
Second highest quantile (5.0–6.99)	1.12	(1.07–1.18)		1.10	(1.00–1.21)		1.14	(1.09–1.19)	
Highest quantile (≥7.0)	Reference			Reference			Reference		
Missing	1.08	(1.01–1.17)		1.19	(1.04–1.37)		1.16	(1.08–1.25)	

aOR, adjusted odds ratio; CI, confidence interval.

^a^Adjusted for respondent of the survey, caregivers of the child, cohort generation, gender of the child, presence of siblings of the child, living in a two-parent household, living in a three-generation household, parent’s age, education, working hours, work type, family income, and always use of other forms of discipline.

Table 4 shows the association between risk factors and spanking, stratified by cohort generation. In general, the associations were similar in both cohorts. However, the likelihood of spanking was lower when both parents were the respondents to the survey only in the first cohort. On the contrary, the associations with spanking in the second cohort were more evident when other family members were respondent, for the presence of a sibling of the child, parents’ lower level of education, and parents’ work types (no outside work, employed part-time, self-employed, domestic side job, and others).

**Table 4.** Adjusted^a^ odds ratios for spanking stratified by cohort generation

	First cohort						Second cohort					
	
	Population at risk	Case		aOR	(95% CI)	Trend *P*	Population at risk	Case		aOR	(95% CI)	Trend *P*
	
		Sometimes spanking	Always spanking					Sometimes spanking	Always spanking			
	41,193	27,727	4,075				29,257	16,544	1,523			
Respondents of the survey												
Mother	38,044	25,714	3,829	Reference		0.001	24,305	13,958	1,266	Reference		0.07
Father	2,207	1,391	166	0.88	(0.79–0.98)		1,933	889	107	0.82	(0.74–0.92)	
Both	603	404	50	0.79	(0.66–0.93)		2,687	1,506	132	0.96	(0.88–1.04)	
Other family members	339	218	30	1.23	(0.96–1.58)		332	191	18	1.45	(1.15–1.84)	
Gender of the child												
Boy	21,399	14,880	2,484	1.49	(1.43–1.55)	<0.0001	15,066	9,027	965	1.46	(1.40–1.54)	<0.0001
Girl	19,794	12,847	1,591	Reference			14,191	7,517	558	Reference		
Presence of siblings of the child												
No	9,966	6,328	839	Reference		<0.0001	6,950	3,379	267	Reference		<0.0001
Yes	31,227	21,399	3,236	1.31	(1.25–1.38)		22,307	13,165	1,256	1.52	(1.44–1.61)	
Living in a two-parent household												
No	2,060	1,350	244	1.16	(1.04–1.29)	0.01	1,240	706	92	1.25	(1.09–1.42)	0.0004
Yes	39,133	26,377	3,831	Reference			28,017	15,838	1,431	Reference		
Living in a three-generation household												
No	32,889	22,412	3,233	1.24	(1.18–1.31)	<0.0001	24,805	14,103	1,280	1.24	(1.16–1.33)	<0.0001
Yes	8,304	5,315	842	Reference			4,452	2,441	243	Reference		
Parent’s age, years												
<25	930	591	153	1.16	(1.00–1.35)	<0.0001	354	218	32	1.12	(0.89–1.41)	<0.0001
25–29	6,865	4,604	922	1.13	(1.07–1.21)		3,361	2,014	289	1.16	(1.06–1.26)	
30–34	17,609	11,973	1,728	Reference			9,646	5,515	510	Reference		
35–39	11,829	7,958	1,000	0.96	(0.91–1.01)		10,493	5,937	471	0.97	(0.92–1.03)	
40–44	3,158	2,070	217	0.85	(0.78–0.92)		4,437	2,359	173	0.89	(0.83–0.96)	
≥45	463	313	25	0.89	(0.73–1.09)		634	310	30	0.89	(0.75–1.05)	
Parent’s education												
Junior high school	1,376	908	204	1.55	(1.36–1.77)	<0.0001	778	459	77	1.65	(1.40–1.93)	<0.0001
High school	15,413	10,540	1,831	1.53	(1.43–1.64)		7,339	4,417	511	1.54	(1.44–1.65)	
Vocational	7,574	5,231	765	1.46	(1.36–1.57)		5,476	3,283	314	1.48	(1.38–1.59)	
Junior college	9,224	6,242	757	1.24	(1.15–1.32)		5,989	3,446	261	1.30	(1.21–1.39)	
University and higher	6,283	3,936	381	Reference			8,180	4,078	277	Reference		
Others	39	27	2	0.91	(0.47–1.79)		82	40	5	0.92	(0.59–1.45)	
Missing	945	625	105	1.36	(1.17–1.58)		1,081	630	60	1.38	(1.21–1.58)	
Parent’s work type												
No outside work	20,311	13,919	2,091	1.18	(1.00–1.38)	0.95	11,426	6,765	589	1.25	(1.10–1.41)	0.38
Seeking employment	1,716	1,124	196	1.08	(0.89–1.3)		1,243	682	83	1.13	(0.96–1.34)	
Students	71	47	4	0.87	(0.51–1.47)		38	22	4	2.05	(1.04–4.02)	
Employed full-time	7,785	4,988	634	Reference			7,962	4,083	342	Reference		
Employed part-time	7,054	4,826	722	1.06	(0.97–1.16)		5,721	3,358	337	1.13	(1.03–1.23)	
Self-employed	2,324	1,536	221	1.05	(0.94–1.17)		1,408	796	69	1.17	(1.04–1.33)	
Domestic side job	810	563	90	1.12	(0.94–1.32)		284	163	27	1.33	(1.03–1.72)	
Others	374	231	45	1.07	(0.85–1.34)		330	193	23	1.40	(1.10–1.78)	
Missing	409	275	42	1.14	(0.85–1.53)		513	291	31	1.16	(0.92–1.47)	
Family income (1,000,000 JPY)												
Lowest quantile (0–<3.8)	9,918	6,717	1,214	1.23	(1.15–1.32)	<0.0001	6,060	3,640	392	1.25	(1.16–1.36)	<0.0001
Second lowest quantile ​ (3.8–4.99)	8,287	5,709	874	1.21	(1.13–1.29)		5,510	3,296	326	1.30	(1.20–1.40)	
Second highest quantile ​ (5.0–6.99)	11,020	7,502	1,020	1.12	(1.05–1.19)		8,036	4,581	406	1.17	(1.09–1.25)	
Highest quantile (≥7.0)	9,312	6,024	677	Reference			7,575	3,860	256	Reference		
Missing	2,656	1,775	290	1.16	(1.05–1.28)		2,076	1,167	143	1.16	(1.04–1.29)	

aOR, adjusted odds ratio; CI, confidence interval.

^a^Adjusted for respondent of the survey, caregivers of the child, cohort generation, gender of the child, presence of siblings of the child, living in a two-parent household, living in a three-generation household, parent’s age, education, working hours, work type, family income, and always use of other forms of discipline.

Table 5 shows the associations between risk factors and spanking, stratified by gender of the child. The associations were generally similar in both genders. However, the likelihood of spanking was lower in girls when the father was the respondent to the survey.

**Table 5.** Adjusted^a^ odds ratios for spanking stratified by gender of the child

	Boys						Girls					
	
	Population at risk	Cases		aOR	(95% CI)	Trend *P*	Population at risk	Cases		aOR	(95% CI)	Trend *P*
	
		Sometimes spanking	Always spanking					Sometimes spanking	Always spanking			
	41,193	27,727	4,075				29,257	16,544	1,523			
Respondents of the survey												
Mother	32,158	21,317	3,124	Reference		0.06	30,191	18,355	1,971	Reference		0.004
Father	2,212	1,308	169	0.91	(0.82–1.01)		1,928	972	104	0.79	(0.71–0.89)	
Both parents	1,761	1,075	125	0.93	(0.84–1.03)		1,529	835	57	0.91	(0.82–1.02)	
Other family members	334	207	31	1.41	(1.10–1.80)		337	202	17	1.26	(0.99–1.60)	
Cohort Generation												
First	21,399	14,880	2,484	Reference		<0.0001	19,794	12,847	1,591	Reference		<0.0001
Second	15,066	9,027	965	0.54	(0.51–0.56)		14,191	7,517	558	0.55	(0.52–0.57)	
Presence of siblings of the child												
No	8,654	5,211	671	Reference		<0.0001	8,262	4,496	435	Reference		<0.0001
Yes	27,811	18,696	2,778	1.43	(1.35–1.50)		25,723	15,868	1,714	1.37	(1.30–1.44)	
Living in a two-parent household												
No	1,705	1,091	186	1.12	(1.00–1.25)	0.15	1,595	965	150	1.27	(1.13–1.42)	<0.0001
Yes	34,760	22,816	3,263	Reference			32,390	19,399	1,999	Reference		
Living in a three-generation household												
No	29,878	19,751	2,771	Reference		<0.0001	27,816	16,764	1,742	Reference		<0.0001
Yes	6,587	4,156	678	0.82	(0.77–0.87)		6,169	3,600	407	0.79	(0.75–0.84)	
Parent’s age, years												
<25	334	207	31	1.17	(0.99–1.40)	<0.0001	337	202	17	1.13	(0.94–1.34)	<0.0001
25–29	657	427	103	1.11	(1.03–1.19)		627	382	82	1.18	(1.10–1.26)	
30–34	5,329	3,550	713	Reference			4,897	3,068	498	Reference		
35–39	14,203	9,472	1,396	0.95	(0.90–1.00)		13,052	8,016	842	0.98	(0.93–1.04)	
40–44	11,462	7,462	923	0.89	(0.82–0.96)		10,860	6,433	548	0.85	(0.78–0.92)	
≥45	3,901	2,433	255	0.83	(0.70–0.99)		3,694	1,996	135	0.95	(0.78–1.14)	
Parent’s education												
Junior high school	1,101	730	162	1.65	(1.43–1.90)	<0.0001	1,053	637	119	1.53	(1.33–1.77)	<0.0001
High school	11,768	7,985	1,405	1.56	(1.46–1.67)		10,984	6,972	937	1.53	(1.43–1.63)	
Vocational	6,824	4,631	682	1.54	(1.44–1.66)		6,226	3,883	397	1.41	(1.31–1.52)	
Junior college	7,871	5,223	657	1.29	(1.20–1.38)		7,342	4,465	361	1.25	(1.16–1.34)	
University and higher	7,460	4,416	412	Reference			7,003	3,598	246	Reference		
Others	60	36	3	0.83	(0.49–1.41)		61	31	4	1.05	(0.62–1.78)	
Missing	1,047	679	97	1.37	(1.19–1.57)		979	576	68	1.38	(1.20–1.59)	
Parent’s work type												
No outside work	16,390	11,137	1,672	1.23	(1.07–1.41)	0.70	15,347	9,547	1,008	1.19	(1.04–1.37)	0.62
Seeking employment	1,588	1,010	174	1.09	(0.92–1.29)		1,371	796	105	1.11	(0.94–1.32)	
Students	57	37	4	0.98	(0.55–1.73)		52	32	4	1.43	(0.80–2.57)	
Employed full-time	8,173	4,982	599	Reference			7,574	4,089	377	Reference		
Employed part-time	6,630	4,381	626	1.03	(0.94–1.13)		6,145	3,803	433	1.17	(1.07–1.29)	
Self-employed	1,957	1,271	173	1.08	(0.96–1.21)		1,775	1,061	117	1.14	(1.02–1.29)	
Domestic side job	520	359	72	1.29	(1.05–1.59)		574	367	45	1.12	(0.93–1.36)	
Others	357	229	48	1.45	(1.15–1.84)		347	195	20	1.04	(0.83–1.31)	
Missing	459	294	50	1.23	(0.96–1.59)		463	272	23	1.09	(0.85–1.39)	
Family income (1,000,000 JPY)												
Lowest quantile (0–<3.8)	8,262	5,533	943	1.23	(1.14–1.32)	<0.0001	7,716	4,824	663	1.26	(1.17–1.36)	<0.0001
Second lowest quantile ​ (3.8–4.99)	7,138	4,807	742	1.23	(1.15–1.32)		6,659	4,198	458	1.26	(1.17–1.36)	
Second highest quantile ​ (5.0–6.99)	9,934	6,573	902	1.13	(1.06–1.21)		9,122	5,510	524	1.15	(1.08–1.23)	
Highest quantile (≥7.0)	8,712	5,400	600	Reference			8,175	4,484	333	Reference		
Missing	2,419	1,594	262	1.21	(1.09–1.35)		2,313	1,348	171	1.11	(1.00–1.23)	

aOR, adjusted odds ratio; CI, confidence interval.

^a^Adjusted for respondent of the survey, caregivers of the child, cohort generation, gender of the child, presence of siblings of the child, living in a two-parent household, living in a three-generation household, parent’s age, education, working hours, work type, family income, and always use of other forms of discipline.

## DISCUSSION

In this study using national longitudinal survey data, we present an overview of family members’ spanking of 3.5-year-old children and the associated factors. We found more than 70% of family members spanked their children, which was consistent with previous reports; corresponding rates exceed 70% in some European, Asian, and African countries.^13^^,^^21^ We found that more children in the second cohort investigated in 2013 were never spanked (38%) compared with those in the first cohort investigated in 2004–2005 (23%). The increase in the prevalence of never spanking among the second cohort might reflect greater social awareness of child abuse. Even though spanking and corporal punishment have not been banned in Japan in the investigated periods, the Child Welfare Act (Act No. 164 of 1947)^15^ and the Act on the Prevention, etc of Child Abuse (Act No. 82 of 2000)^16^ were amended several times during the interval between the survey waves. In fact, the substantial increase in the annual number of reported cases of suspected child abuse to child guidance centers increased from 17,725 cases in 2000, to 33,408 in 2004, and to 88,931 in 2014.^23^

The cohort generations modified the associations between parents’ level of education or parents’ work type and spanking. This may reflect changes in the distribution and nature of these variables over time, as more parents shifted to higher education and engaged in full-time employment in the second cohort. These changes over generations also reflected the fact that, if the respondents of the survey were other family members (ie, mostly grandparents), they were more likely to spank the children than in families where mothers were the respondents.

In this study, if the respondents of the survey were fathers or both parents, they were less likely to spank their children than in families where the respondents were mothers. This finding was consistent with the results of previous studies in the United States and Hong Kong.^4^^,^^10^^,^^24^^,^^25^ indicating that fathers spanked less frequently than mothers, although this was considered a consequence of mothers typically spending more time with children than fathers.^10^^,^^24^ However, a previous study using the same longitudinal survey data as the present study showed that the father’s involvement in childcare prevented unintentional injuries,^26^ implying that the father’s involvement in parenting may be beneficial for adverse child outcomes. It may also be because responses to the survey by both parents may reflect a good marital relationship, which is beneficial for refraining from spanking.^27^

Consistent with previous studies in the United States,^1^^,^^24^ being a boy was a risk factor of being spanked for misbehavior. This is probably due to the different types of misbehaviors and parents’ reactions by gender.^10^ The analysis stratified by the child’s gender showed that being a girl was a modifier for the father’s spanking reaction to misbehavior.

Regarding family structure, the presence of siblings of the child and not living in a two-parent household were risk factors of being spanked, which was also consistent with previous findings.^1^^,^^10^ We also found that living in a three-generation household was a protective factor of spanking, which could be due to informal social support for parents from family members and greater assistance with household chores.^10^^,^^28^^,^^29^ Regarding socioeconomic factors, our results found that a lower level of parental education or family income were risk factors of spanking, which was consistent with the findings of previous reports in the United Kingdom and United States.^10^^,^^30^ We found that, in addition to unstable work types, such as part-time employment, self-employment, and domestic side jobs, no outside work (ie, housewife) was also a risk factor for spanking after adjusting for other socioeconomic factors. This is consistent with a previous study of 1,662 participants in Hong Kong showing the association between the respondent’s (mother’s or father’s) unemployment and corporal punishments, including spanking.^25^ The reason for this may be mainly because unemployed parents typically spend more time with their children. However, considering the fact that these results were obtained after adjusting for working hours, other assumptions could be added. First, mothers who are employed full-time could have better moods at home compared with non-working mothers.^31^ Second, since full-time employment offers a wider range of social and professional contacts,^32^ parents who are employed full-time could have developed more social skills, including anger management, which help them choose other strategies of child discipline apart from corporal punishment. Third, parents who work full-time may feel guilty about leaving their children to go work, resulting in warmer parenting^33^^,^^34^ to compensate for their absence during working hours compared with non-working parents.

We found that more than half of the family members in this study had spanked their children. It is important to promote greater involvement of fathers in parenting and to educate parents in alternative forms of discipline to handle their child’s misbehavior or conflicting situations in order to prevent or reduce the use of spanking.^35^ For example, the most prevalent reaction under such situations in Sweden is “to divert the child’s attention from the cause of the misbehavior”, followed by “discussion with the child”.^36^ In the United States, “time outs”, which physically remove the child from where he/she is misbehaving, are preferred.^35^ These alternative forms of discipline do not seem to be commonly used in Japan considering the response items in the questionnaire. Parent training programs are one opportunity to provide information on alternative forms of discipline used in other countries. According to a meta-analysis evaluating effective parent training programs, requiring parents to practice with their child during training sessions showed better parent and child outcomes.^37^ Considering our data that more mothers were in the workforce and more fathers were involved in parenting in the second cohort than in the first cohort, providing accessible parenting programs held not only on weekdays, but with flexible participation schedules (eg, on weekends, or as webinars, or even during lunch time at the workplace) could be suggested.

The major strengths of this study include the large population-based sample that consisted of two generation cohorts. Also, the response choices to the question on how to react to child misbehavior comprised five reactions, and respondents were required to indicate the frequency for each reaction, which could reduce underestimation of the prevalence of spanking compared with previous studies that required answering about the frequency of spanking only.^6^ Several limitations should be discussed. First, there might be cultural or ethnic differences regarding the use of spanking. Therefore, it might be difficult to generalize these findings to other populations. Second, some exposure variables were obtained in the same wave survey as the outcome “spanking”; thus, our findings could not confirm a causal association. Third, the outcome “spanking” was not validated or evaluated in an objective manner by referring to the number of times spanking was used during some specific period of time. However, this is difficult in practice because there are no gold standard measurements for the comparison. Fourth, the fathers responded in this survey were limited in number and these subjects may have been biased toward “good fathers”. Therefore, further studies which require father’s and mother’s responses respectively, will be necessary to confirm the protective effect of father’s involvement on spanking. Fifth, residual confounding could have occurred from unmeasured confounding variables. For example, the variables related to parents’ stress or children’s temperaments were not investigated in this study.

In conclusion, our study suggested that spanking is less frequent in more recent generations and the father’s involvement in parenting may be protective against spanking. Moreover, the child’s gender, family structure, and factors of low socioeconomic status including no outside work were associated with spanking.
